# PSMA-guided management of recurrent post-prostatectomy patients: a sub-analysis of a prospective single-center study

**DOI:** 10.3389/fonc.2026.1764588

**Published:** 2026-01-29

**Authors:** Giuseppe Carlo Iorio, Cristiano Grossi, Valeria Chiofalo, Veronica Richetto, Guido Rovera, Diego Bongiovanni, Ramona Parise, Francesca Clot, Marco Oderda, Serena Grimaldi, Erica Maria Cuffini, Mario Levis, Paolo Gontero, Silvia Morbelli, Umberto Ricardi

**Affiliations:** 1 Department of Oncology, School of Medicine, University of Turin, Torino, Italy; 2 Medical Physics Unit, A.O.U. Città della Salute e della Scienza di Torino, Torino, Italy; 3 Department of Nuclear Medicine, AOU Città della Salute e della Scienza di Torino, University of Turin, Turin, Italy; 4 Urology Clinic, Department of Surgical Sciences, City of Health and Sciences at Molinette University Hospital, University of Turin, Turin, Italy

**Keywords:** BCR, biochemical recurrence, PCa, prostate cancer, prostate-specific membrane antigen - positron emission tomography, PSMA-PET, salvage radiotherapy, SRT

## Abstract

**Introduction:**

The advent of prostate-specific membrane antigen - positron emission tomography (PSMA-PET) imaging influences prostate cancer (PCa) salvage radiotherapy (SRT) decision-making, especially in biochemical recurrence (BCR) with low PSA. This study evaluates the impact of PSMA-PET on recurrent post-prostatectomy patients treated with radiotherapy (RT) ± hormonal therapy (HT).

**Materials and methods:**

In a prospective, observational study (2016–2020), 103 hormone-sensitive post-prostatectomy pN0-pNx PCa patients with proven BCR were analyzed. PSMA-positive patients received tailored treatments based on lesion sites, ranging from prostate bed (P-bed) SRT abort, metastases-directed therapy, to systemic therapy. PSMA-negative patients were mainly treated with SRT. Primary objectives included PSMA-based management changes and biochemical progression-free survival (bPFS) comparison.

**Results:**

PSMA-PET was positive in 28.1% (29/103) at a median PSA of 0.5 ng/mL. The most common relapse sites were bones and pelvic lymph nodes. Among positive PSMA-PET patients, the P-bed abort rate was 58.6% (17/29 patients). Excluding patients with PSMA-positive uptake solely in the P-bed (7 patients), the rate of P-bed abort was 77.3% (17/22 patients). Management was altered in 75.8% of PSMA-positive and 21.3% overall. Most PSMA-positive lesions were managed with SBRT, either as a standalone treatment or within combined-modality approaches: SBRT was delivered to 50% of nodal recurrences (including both N1 and M1a cases) and to 100% of bone lesions. At 5 years, bPFS was 66.8% in PSMA-negative patients (undergoing SRT+/-HT) vs. 26.7% in PSMA-positive patients (any treatment).

**Conclusion:**

PSMA-PET led to management changes in nearly one-third of post-prostatectomy recurrent PCa, notably affecting RT strategies and systemic therapy. Emerging evidence suggests that treatment intensification based on PSMA-PET findings may improve patient outcomes compared to de-intensified approaches. The impact on outcomes awaits validation from ongoing prospective randomized trials.

## Introduction

In prostate cancer (PCa) patients treated with radical prostatectomy (RP), particularly those with unfavorable features, biochemical evidence of disease progression or inadequate response to treatment can be observed in 20–40% of cases (1, 2). The ARTISTIC meta-analysis has suggested that adjuvant radiotherapy (ART) following RP does not improve event-free survival (EFS) compared to early salvage treatment (eSRT) in a population of mainly pN0-pNx patients (3). Thus, eSRT seems to be the preferable treatment policy as it offers the opportunity to spare radiotherapy (RT) and its associated side effects for many men in this setting (3). However, pN1 patients with four or more positive pelvic lymph nodes appear to benefit from using ART in terms of decreased mortality (4). In the case of SRT at biochemical recurrence (BCR), the use and exact duration of androgen deprivation therapy (ADT) have still to be fully defined as patient selection remains crucial (5–8). In terms of radiation volumes, the extension of the standard prostate bed (P-bed) field to include the pelvic lymph nodes (when used in combination with short-term ADT) in pN0-pNx patients was found to result in the greatest impact on outcomes in the RTOG 0534 trial (9). However, it is important to note that most of the data available in the literature regarding SRT (+/-ADT) are based on conventional imaging staging and, thus, affecting the optimal characterization of a standard of care (10). Indeed, the recent advent of more prostate-specific positron emission tomography (PET) imaging tracers is set to affect and redefine the clinical practice in the context of SRT decision-making and planning (11, 12). Prospective data showed that prostate-specific membrane antigen (PSMA) should be the tracer of choice when PET imaging is considered for subsequent treatment management decisions in patients with BCR and low prostate-specific antigen (PSA) concentrations (13). Thanks to its higher detection rates, PSMA-PET can lead to management changes in one-third of patients receiving SRT (12). Our prospective study aimed at testing the performance of PSMA-PET imaging in a population of recurrent hormone-sensitive PCa (HSPC) patients candidate for salvage therapy following primary treatment (either RP or RT) (14, 15). Firstly, we reported PSMA-PET overall good detection performance (14) and its prognostic significance since a lower incidence of events was observed in patients having negative scans (15). The present analysis, with extended follow-up, focuses on the post-RP pN0-pNx study population subgroup experiencing BCR and reports on the PSMA impact on subsequent RT management.

## Materials and methods

### Study design and inclusion/exclusion criteria

This is a prospective, open label, observational, single-center study in PCa patients, approved by the local ethical committee of the City of Health and Science University Hospital (University of Turin, Turin, Italy; protocol P-5315). All patients recruited at the uro-oncological tumor board of the City of Health and Science University Hospital signed an informed consent form (ICF) before enrolment. Patients relapsing following RP were investigated (following enrolment) with Gallium-68(^68^Ga)-PSMA-11-PET/computed tomography (CT) at a single referral center (Division of Nuclear Medicine, City of Health and Science University Hospital) between November 2016 and September 2020 (14, 15).

Radiation treatments for the patients included in the present study sub-analysis (both PSMA-positive and PSMA-negative) were performed at the Department of Oncology-Division of Radiation Oncology of the City of Health and Science University Hospital.

Inclusion criteria for the present sub-analysis were (1) previous RP; (2) pN0 or pNx; (3) proven first-time BCR, two consecutive PSA values of ≥0.2 ng/mL (16); (4) hormone-sensitive state. Exclusion criteria were (1) previous ART; (2) ADT following RP prior to the protocol PSMA-PET.

Based on RTOG 0534 data (9), a PSA cutoff of >0.35 ng/mL was applied for the descriptive categorization of patient subgroups, using PSA values available at the time of treatment decision.

At our Institution, the standard treatment for pN0-pNx patients with BCR (and negative imaging) included P-bed SRT, with hormonal therapy (HT) at the clinician’s discretion. Thus, this cluster of patients was treated accordingly in the case of negative protocol PSMA-PET (STD subgroup). However, based on the clinical case and according to clinician discretion, pN0-pNx patients with BCR and negative protocol PSMA scan could also be treated solely with HT or initially observed.

In the presence of a positive PSMA-PET, the therapeutic options allowed were as follows:

- In the case of 1 to 3 pathological uptakes (either regional or distant non-visceral metastases) solely outside of the P-bed: (a) P-bed abort with metastases-directed therapy (MDT), either surgical or stereotactic body RT (SBRT) +/- HT; (b) the addition of MDT to P-bed irradiation +/- HT; (c) HT only. Indeed, regarding MDT, per our Institutional standards, no more than 3 simultaneous lesions (either regional or distant) were considered eligible for SBRT.
- In the case of exclusive P-bed pathological uptake: SRT and eventually, at the clinician’s discretion, (a) radiation dose escalation (boost) and/or (b) HT addition.
- In the case of mixed-site pathological uptakes (P-bed and 1–3 uptakes outside of the P-bed): (a) SRT+SBRT+/-HT or (b) HT only;
- In the case of >3 PSMA-positive distant metastatic sites or visceral involvement: (a) only systemic therapy was allowed.

### Objectives

The primary objective was to assess the rate of PSMA-based changes in the clinical management (CiM) in patients with positive scans, namely any treatment different from STD approach (CiM subgroup): P-bed abort with MDT (+/-HT), SRT/MDT treatment intensification (SRT plus MDT+/-HT) or intensified (in terms of length and/or agents’ combination) systemic treatment alone.

P-bed pathological PSMA uptakes eventually leading (at the clinician’s discretion) to SRT dose escalation (boost) and/or HT addition were not considered for the CiM rate.

Biochemical progression-free survival (bPFS) comparison between the PSMA-positive (any treatment) and -negative (SRT+/-HT) subgroups was included as a primary objective (Kaplan-Meier survival curves).

Secondary objectives were:

- PSA kinetics analysis for both PSMA positive and negative scans.
- Patterns of failure for each treatment subgroup: (a) relapse site; (b) polymetastatic progression occurrence (>3 distant metastatic lesions).

Additionally, in the present analysis we report SBRT-MDT related toxicity events, scored according to the Common Terminology Criteria for Adverse Events (CTCAE) version 4.03 (v4.03).

Median follow-up was evaluated from the protocol PSMA-PET date to the last patient evaluation date (either follow-up visit or death).

### Outcomes measurements and statistical analysis

For bPFS assessment, biochemical progression following P-bed irradiation (+/-HT) was considered in the case of PSA ≥0.4 ng/mL and rising after treatment completion (3). This definition was also employed for the CiM subgroup in the case of SRT+SBRT (+/-HT). For bPFS assessment, biochemical progression following P-bed abort and SBRT (+/- HT) was defined as a PSA increase above 0.2 ng/mL for patients with a PSA nadir < 0.2 ng/mL or 2 consecutive PSA increases > 25% if compared to nadir in patients with a PSA nadir > 0.2 ng/mL. In patients with no ongoing HT, anticipated restaging (either PSMA or conventional imaging) was allowed at the clinician’s discretion (e.g., PSA kinetics or suspicion of symptomatic progression). In case of CRPC occurrence, conventional imaging was performed.

All *p* values were obtained by the two-sided exact method at the conventional 5% significance level.

Data were analyzed by MedCalc Statistical Software *v.15.8* (MedCalc Software, Ostend, Belgium).

### Radiotherapy, radiopharmaceuticals and PET/CT imaging procedures

P-bed RT delivery was performed through either 3D conformal RT (3DCRT) or volumetric arc therapy (VMAT), employing the Elekta Sinergy or Axesse LINAC systems (Elekta, Stockholm, Sweden). During the accrual period of the study, the standard doses (no boost) for P-bed SRT at our Institution were: 70 Gy in 35 fractions or 52.5 Gy in 20 fractions, at the clinician’s discretion. In the case of MDT, VMAT-based SBRT delivery was performed (1 to 5 fractions) employing exclusively the Elekta Axesse LINAC system (Elekta, Stockholm, Sweden).

The HT regimens (first generation ADT or first-generation peripheral anti-androgens) administered in combination with either SRT or SBRT, at the clinician’s discretion, did not include androgen receptor pathway inhibitors (ARPis).

^68^Ga-PSMA-11-PET/CT scans were performed at our Division of Nuclear Medicine. ^68^Ga-PSMA-11 was synthesized in the radiochemistry laboratory of our Division of Nuclear Medicine. Gallium-68 was produced with a ^68^Ge/^68^Ga generator (ITG Isotope Technologies Garching GmbH, Germany). All patients received an intravenous dose of 2 MBq/Kg ± 0.2 MBq of ^68^Ga-PSMA-11 followed by intravenous hydration (0.5 L, saline solution) during uptake, as previously reported (14, 15). No specific patient preparation was needed before the procedure, and furosemide or oral contrast media were not administrated. All patients underwent PET/CT imaging with a dedicated tomograph (Gemini Dual, Philips HealthCare). An attenuation-corrected whole-body scan (vertex to mid thighs, 2.5 min per bed position, axial field-of-view of 18 cm per bed position) was acquired 60 minutes after tracer injection. A low-dose CT scan was performed for attenuation correction of the PET emission data. The PET scans were reconstructed using ordered subset expectation maximization (OSEM)–based algorithms. Further details on radiopharmaceuticals and PET/CT imaging procedures have been previously published (14, 15).

## Results

103 patients were included in the present sub-analysis. The median follow-up was 64.7 months (IQR: 43.6–76 months). Patients with pT3 stage represented the 42.7% of the population. Positive margins (R+) were observed in 35.9% of cases. pN0 patients were 77.7%, while the remaining were pNx. Patients and disease characteristics details are reported in **Table 1**.

**Table 1:** Patients and disease characteristics.

Parameter	Value
Median age at RP	67 years (range: 51–79)
Median age at Protocol PSMA	70 years (range: 52–83)
Median iPSA*	8.2 ng/mL (range: 3.1–145)
<pT3	n = 59 (57.3%)
pT3	n = 44 (42.7%)
pN0	n = 80 (77.7%)
pNx	n = 23 (22.3%)
Median N° of removed Nodes**	14 (range: 4–50)
R0	n = 54 (52.4%)
R+	n = 37 (35.9%)
NA R status	n = 12 (11.7%)
ISUP 1	n = 14 (13.6%)
ISUP 2	n = 25 (24.2%)
ISUP 3	n = 36 (35.0%)
ISUP 4	n = 14 (13.6%)
ISUP 5	n = 14 (13.6%)
BCP***	n = 28 (27.1%)

RP, radical prostatectomy; iPSA, initial PSA; PSMA, prostate-specific membrane antigen; NA, not available; ISUP, International Society of Urological Pathology (grade groups, 1-5); BCP, biochemical persistence; R, surgical margins; R0, negative margins; R+, positive margins; N°, number; n, number of patients with a specific disease characteristic.

*iPSA available for 89 patients; **number of removed lymph nodes available in 56 patients; ***BCP defined as PSA ≥ 0.1 ng/mL 4–6 weeks post RP.

The interval between RP and BCR was > 12 months in 62.1% of cases. The median PSA value at the time of first-time BCR was 0.3 ng/mL (IQR: 0.25-0.4 ng/mL). The median PSA value at the time of the protocol PSMA-PET was 0.5 ng/mL (IQR: 0.35-0.83 ng/mL), with a median interval between BCR and PSMA-PET of 3 months (IQR: 1.71-10.44 months).

The details regarding relapse characteristics are reported in **Table 2**.

**Table 2:** Relapse characteristics.

Parameter	Value
Median PSA at BCR after RP	0.3 ng/mL [IQR: 0.25 - 0.405 ng/mL]
Median PSA at protocol PSMA - PSMA negative	0.43 ng/mL [IQR: 0.32 - 0.64 ng/mL]
Median PSA at protocol PSMA - PSMA positive	0.78 ng/mL [IQR: 0.49 - 1.18 ng/mL]
Positive PSMA	n = 29 (28.2%)
Negative PSMA	n = 74 (71.8%)
PSA at treatment – PSMA negative	
Patients with PSA > 0.35 ng/mL	n = 53 (71.6%)
PSA at treatment – PSMA positive	
Patients with PSA > 0.35 ng/mL	n = 27 (93.1%)

RP, radical prostatectomy; PSMA, prostate-specific membrane antigen; BCR, biochemical recurrence; n, number of patients with a specific disease characteristic; IQR, interquartile range.

The protocol PSMA-PET was positive in 29 patients (28.1%), who had a median PSA doubling time (PSAdt) of 6.2 months before the exam (IQR: 2.6 - 10.9).

The median number of positive lesions was 1. The most frequent sites of relapse were: bones (10 patients, 34.4%) and pelvic lymph nodes (9 patients, 31%). No visceral involvement was detected by the protocol PSMA-PET.

However, a patient with 2 occipital bone metastases (and a simultaneous P-bed uptake) underwent further evaluation with magnetic resonance imaging (MRI), which revealed visceral involvement (brain). The latter was also the only case of mixed relapse sites involving both P-bed and extra P-bed uptakes.

**Figure 1:**
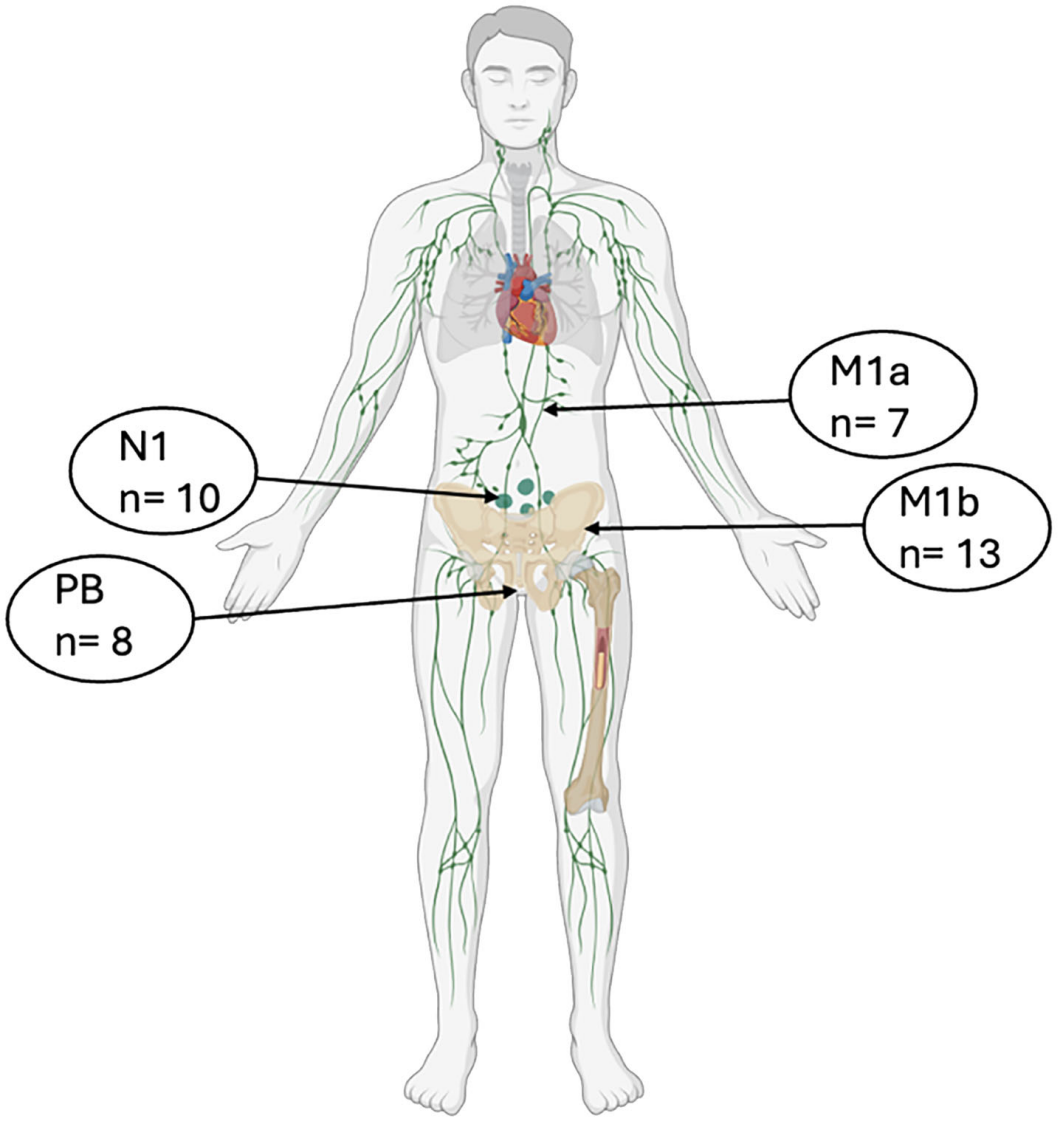
Sites of disease relapse detected at protocol PSMA-PET. n, number of lesions; PB, prostate bed.

A PSMA-positive uptake solely in the P-bed was reported for 7 patients. Sites of relapse details are reported in **Figure 1**.

Among positive PSMA-PET patients, the P-bed abort rate was 58.6% (17/29 patients). Excluding patients with PSMA-positive uptake solely in the P-bed (7 patients), the rate of P-bed abort was 77.3% (17/22 patients). The CiM rate was 75.8% (22/29 patients) among PSMA-PET positive patients and 21.3% (22/103) considering the whole study population.

SBRT alone was the treatment of choice in the 13.8% of PSMA-positive cases (4/29). Details regarding SBRT doses are reported in **Supplementary Table 1**.

**Figure 2:**
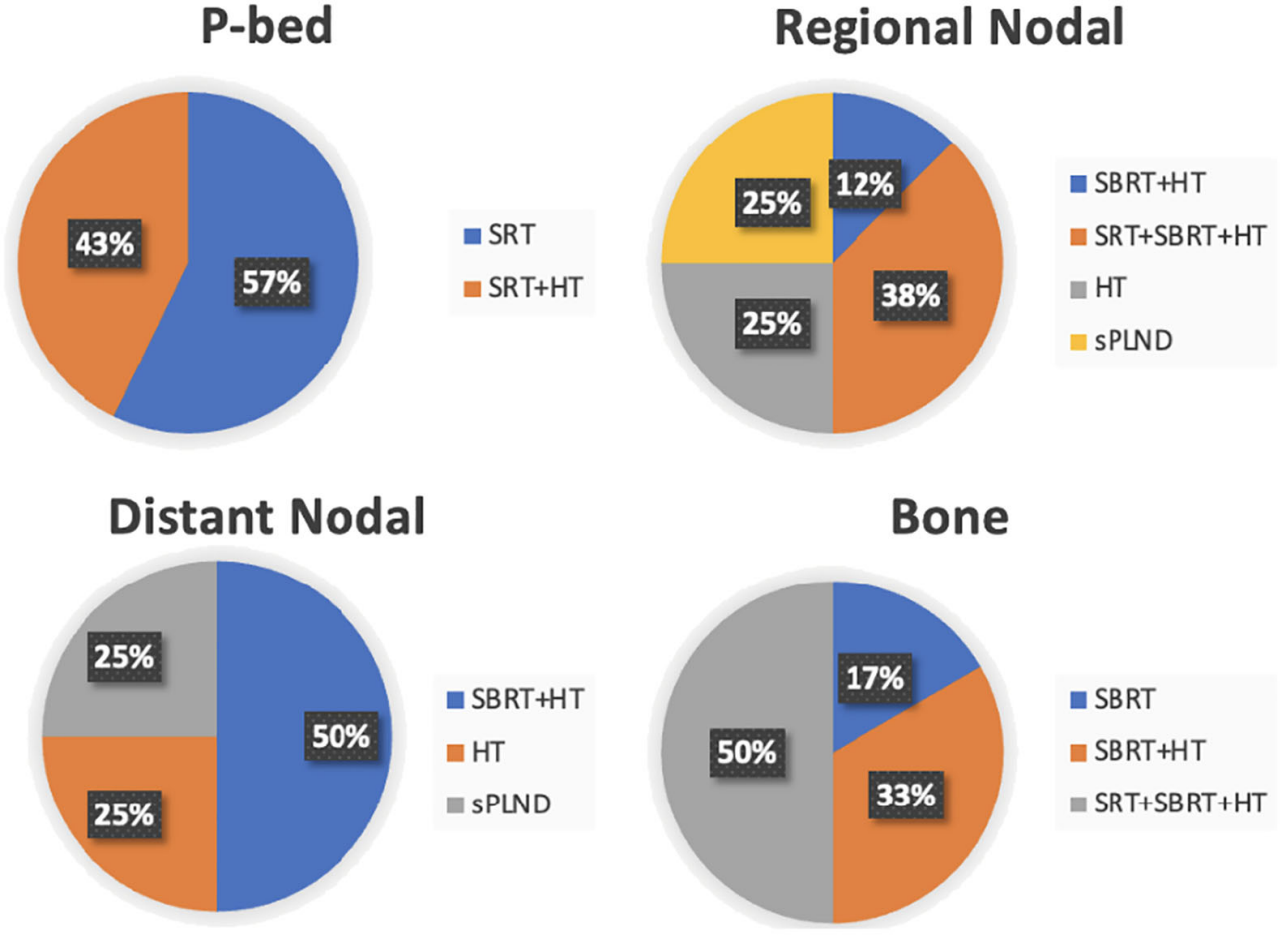
Treatment details stratified by site of recurrence in PSMA-positive patients. P-bed, prostate bed; SRT, salvage radiotherapy; HT, hormonal therapy*; SBRT, stereotactic body radiotherapy; sPLND, salvage pelvic lymph node dissection. *The HT regimens administered in combination with either SRT or SBRT did not include androgen receptor pathway inhibitors (ARPis).

The single case with visceral involvement was treated with HT plus chemotherapy. Treatment details of PSMA-positive patients are illustrated in **Figure 2**.

Except one late G3 pain toxicity (rib metastasis treatment), no other G2+ were reported for patients undergoing SBRT-MDT (**Supplementary Table 2**).

Among PSMA-PET negative patients (n=74): 85.2% of cases were treated with P-bed SRT (n=63, with the addition of HT in 5/63 patients); among the remaining ones, 10 patients were observed and 1 was treated with HT alone.

Details regarding SRT doses are reported in **Supplementary Table 1**.

Within the cluster of PSMA-negative patients, PSA at the time of treatment decision was >0.35 ng/mL in 71.6% of cases (53 out of 74 patients). In the latter setting, 81.1% (43 out of 53 patients) received P-bed SRT (with the addition of HT in 2 out of 43 cases).

Among PSMA-PET negative patients, the PSAdt assessed over the 6 months preceding imaging was ≤ 9 months in 64.6% of cases.

At five years follow-up, bPFS rates were 26.7% and 66.8% for PSMA-positive (any treatment) and PSMA-negative undergoing SRT+/-HT patients, respectively (p = 0.0008).

One patient per subgroup was excluded from the bPFS analysis: a PSMA-positive (M1a disease) patient discontinued (poor compliance) the SBRT treatment (combined with HT) and was then lost prior to the first follow-up; a PSMA-negative patient completed the SRT treatment but was then lost prior to the first follow-up.

**Figure 3:**
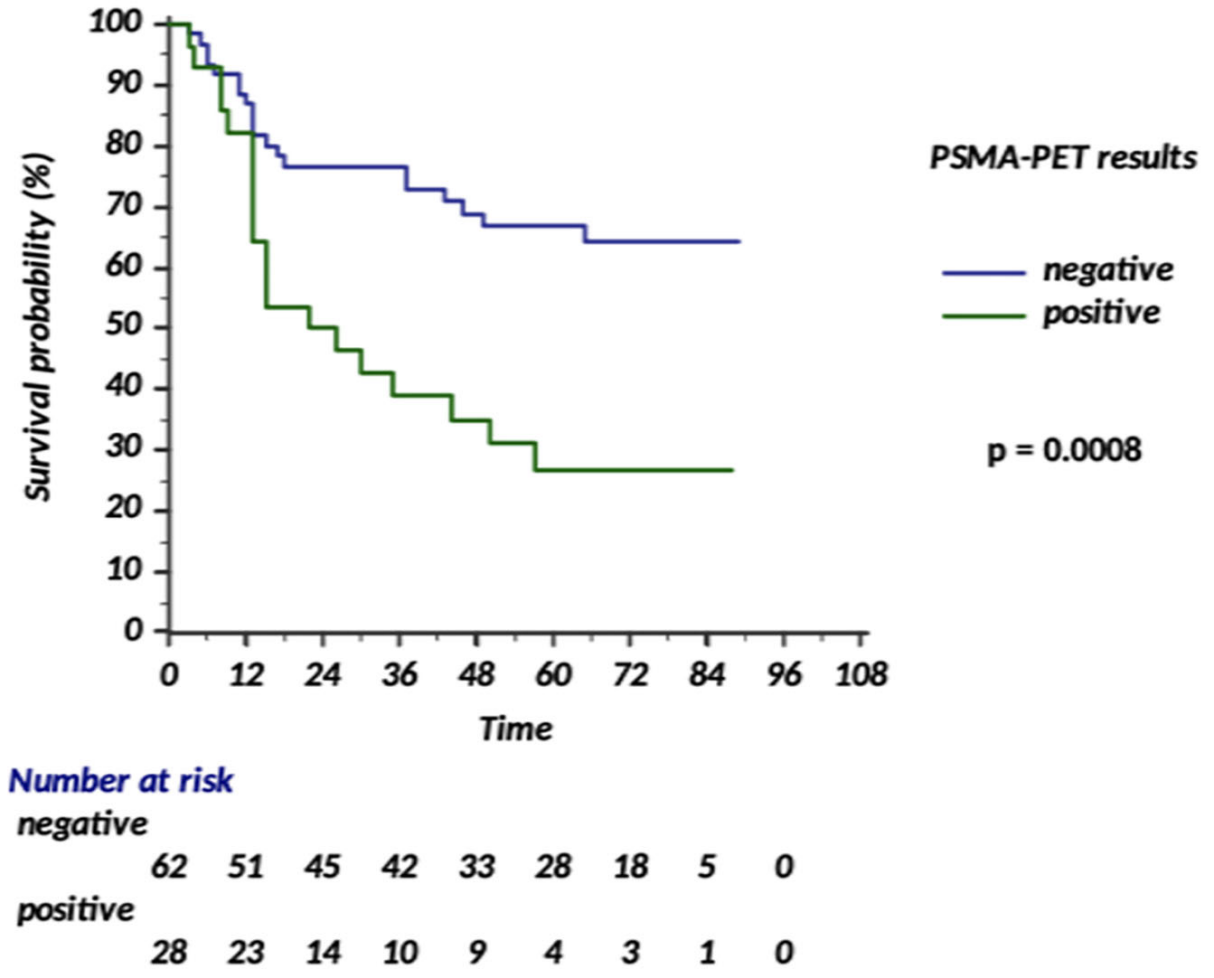
bPFS of PSMA-positive (any treatment) and PSMA-negative (SRT+/-HT) subgroups during the follow-up.

**Figure 3** depicts bPFS of these two subgroups across the different follow-up timepoints.

No P-bed relapse following P-bed abort was observed in PSMA-positive patients with no uptake in the P-bed (0/16).

**Figure 4:**
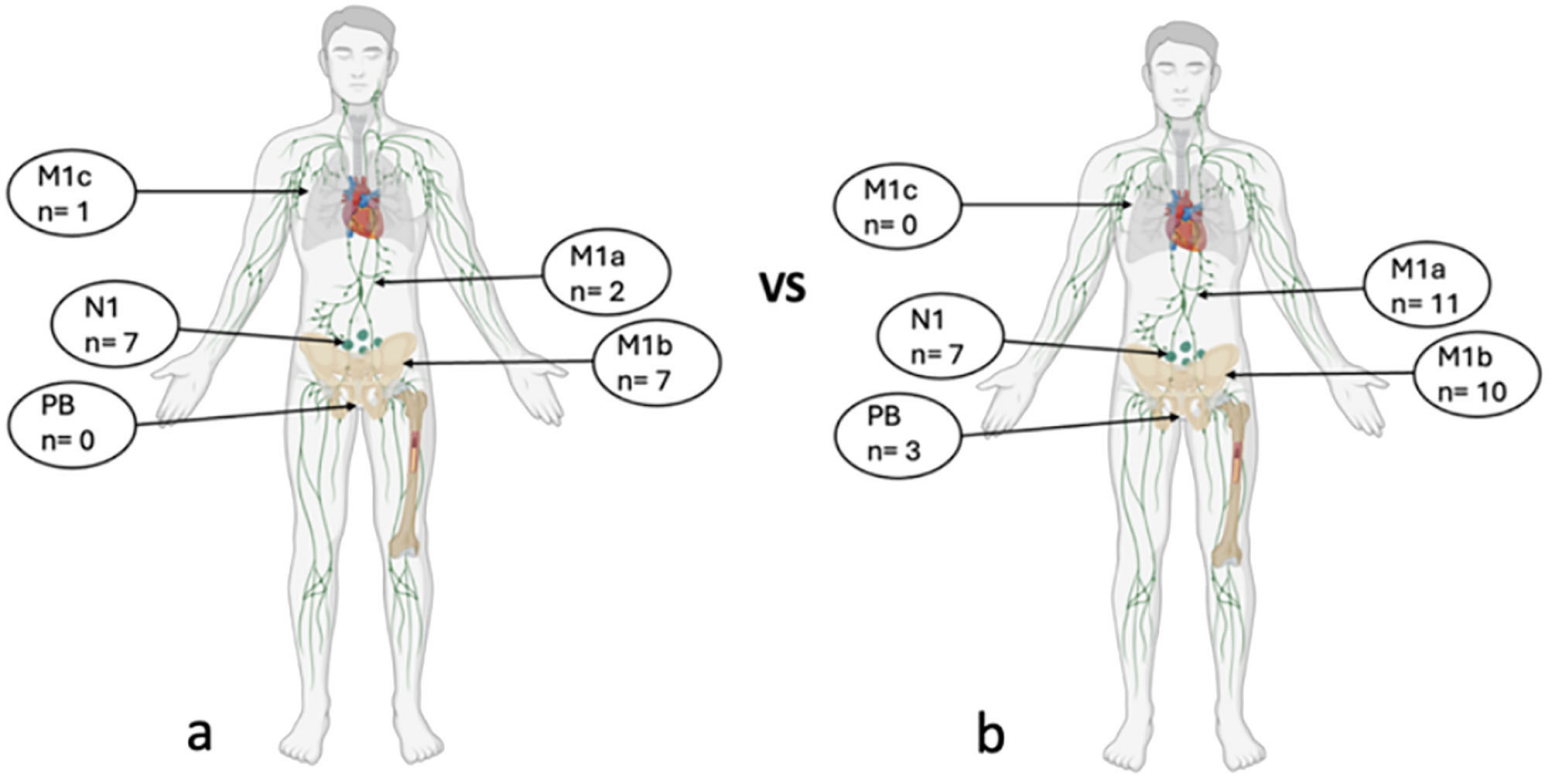
Imaging-detected failure sites following treatment in protocol PSMA-positive patients **(a)** vs protocol PSMA-negative patients **(b)**. n, number of lesions; PB, prostate bed.

Patterns of failure according to the site of recurrence following treatment for both protocol PSMA-positive and -negative subgroups are depicted in **Figure 4**. In 14 cases (7 per subgroup, excluded from **Figure 4**), biochemical progression alone was observed, with no imaging findings.

Following treatment, among protocol PSMA-positive patients no cases of polymetastic progression were observed. One case of polymetastic progression was reported in the protocol PSMA-negative population.

## Discussion

In this sub-analysis of our prospective series (14, 15), we aimed to evaluate the impact of including PSMA-PET imaging into the decision-making process for RT after RP. Indeed, recent data support the use of novel prostate-specific PET tracers (so-called “molecular imaging”) to guide SRT decision-making and planning (11, 12). In the EMPIRE-1 trial, patients with detectable PSA post-RP and negative conventional imaging (no extra-pelvic or bone findings) were randomized to RT directed by conventional imaging alone (Arm 1) vs conventional imaging plus ^18^F-fluciclovine-PET (Arm 2) (11). In Arm 2, RT decisions were rigidly determined by PET, which was also used for target delineation. A dose escalation to sites of uptake strategy was not employed in this trial. PET findings resulted in a 35.4% rate of decision changes, including 4 patients having RT aborted. Median follow-up was 3.52 years. Three-year failure-free survival rate for Arm 1 vs Arm 2 was 63% vs 75.5% (p=0.0028) and at 4-years was 51.2% vs 75.5% (p<0.0001) (11). The phase III PSMA-SRT trial randomized 193 patients with BCR following RP to SRT (control arm, n = 90) or to PSMA prior to SRT planning (investigational arm, n = 103) from June 2018 to August 2020 (12). Any other approved imaging modalities were allowed in both arms. Median PSA levels at enrollment was 0.30 ng/mL in the control arm and 0.23 ng/mL in the PSMA arm. Fluciclovine-PET was used in 43% of cases in the control arm. The rate of PSMA localized recurrences was 38/102 (37%): 9% M1 disease, 16% in the pelvic lymph nodes (N1, with or without local recurrence), and 13% in the P-bed only. There was a 23% difference (p = 0.002) of frequency of major changes between the control arm (22%) and the PSMA arm (45%). There was a 17.6% difference (p=0.005) of treatment escalation frequency between the control arm (12%) and the intervention arm (29%). The final readout of the primary endpoint is expected soon. PET-guided dose-escalation data from the phase II EMPIRE-2 trial (NCT03762759) were recently presented at ASCO GU 2025. This study randomized patients with detectable PSA (and negative conventional imaging) following RP to receive RT guided by either fluciclovine PET (Arm 1) or PSMA PET (Arm 2). There was no significant difference between Arm 1 and Arm 2, with 2-years failure-free survival rates of 88.2% and 86.9%, respectively (p=0.604).

In our study (2016–2020), the median PSA value at the time of first-time BCR was 0.3 ng/mL, and the median PSA value at the time of the protocol PSMA-PET was 0.5 ng/mL (with a median interval between BCR and PSMA-PET of 3 months). The protocol PSMA-PET was positive in 29 patients (28.1%). These patients had a median PSA of 0.78 ng/mL at the time of the protocol PSMA-PET and a PSAdt of 6.2 months prior to the exam, which certainly highlighted an aggressive recurrence cluster.

The median number of positive lesions was 1.

Our primary objective was to assess the rate of PSMA-based CiM, defined as any treatment strategy in patients with positive scans that deviated from the STD post-prostatectomy approach. These included P-bed irradiation omission with MDT (± HT), treatment intensification combining SRT with MDT (± HT), or the use of intensified systemic treatment alone (in terms of treatment duration and/or combination of agents).

Among PSMA-positive, the P-bed abort rate was 58.6% (17/29 patients). Excluding patients with PSMA-positive uptake solely in the P-bed (7 patients), the rate of P-Bed abort was 77.3% (17/22 patients). The CiM rate was 75.8% (22/29 patients) among PSMA positive patients and 21.3% (22/103) considering the whole study population.

Regarding literature data on recurrent regional disease, the recently published PEACE V–STORM phase 2 trial randomized patients with PET-detected pelvic nodal oligorecurrence (up to five nodes following radical local treatment) to MDT (plus 6 months of ADT) or elective nodal RT (ENRT, plus 6 months of ADT) (17). Overall, 24 (25%) of 97 patients in the MDT group and 38 (41%) of 93 in the ENRT group received P-bed RT. In the *post-hoc* analysis, patients receiving (prostate and) P-Bed RT throughout their disease history were less likely to develop a local recurrence than those without (prostate and) P-bed RT (p=0.023). A local recurrence was observed in nine (18%) of 50 patients who never received prostate or P-bed RT versus ten (7%) of 140 patients who did receive prostate or P-bed RT in their disease history (17). In our study, no P-bed relapse following P-bed abort was observed in PSMA-positive patients with no uptake in the P-bed (0/16). However, these findings should be interpreted with caution due to the limited sample size within this subgroup, rendering the analysis underpowered. Specifically designed, large prospective clinical trials are required to determine whether P-bed omission based on PSMA findings can be routinely recommended.

Nonetheless, it is worth noting that the PEACE V-STORM trial showed that P-bed RT was the primary contributor to toxicity (17).

Moreover, the 4-year metastasis-free survival (MFS) was 63% in the MDT group and 76% in the ENRT group (p=0.063). By showing an improved MFS with ENRT, this trial established ENRT as a potential standard treatment approach (17). The PEACE V-STORM trialists, thus, concluded that when MDT is offered for nodal recurrent disease, patients need to be informed that this might result in a higher chance of recurrence and need for subsequent treatments (17). The ongoing POINTER-PC phase 3 trial will aim to confirm these findings (18). Additionally, recent data from a phase 2 randomized clinical trial report improved outcomes with PSMA-PET–guided intensification of SRT including ENRT, nodal boost and MDT (19). In our study, pelvic nodal relapses represented the second most frequent site of recurrence after bone lesions (**Figure 1**). Patients with pelvic nodal recurrence were not offered ENRT. Most PSMA-positive lesions were managed with SBRT, either as a standalone treatment or within combined-modality approaches: SBRT was delivered to 50% of nodal recurrences (including both N1 and M1a cases) and to 100% of bone lesions (**Figure 2**). Intensified treatment strategies combining SRT, SBRT, and HT were used in 38% of regional nodal recurrences and 50% of bone recurrences. At five years of follow-up, bPFS rates were 26.7% for PSMA-positive patients (any treatment) and 66.8% for PSMA-negative patients undergoing SRT+/-HT (**Figure 3**). These findings are consistent with the established prognostic significance of PSMA status, as reported in our earlier analysis (15), since it appears to stratify patients according to disease burden and biological aggressiveness.

The outcome observed in the PSMA-positive subgroup should be interpreted with caution mainly due to the heterogeneity of treatment approaches (based on clinician discretion) and the limited number of patients within each recurrence site category. Consequently, within the PSMA-positive cluster, any analysis of differential outcomes across the different treatment modalities would have been inappropriate, as it would not have been possible to causally attribute the potentially observable differences to specific interventions. Rather, such differences would have more likely reflected variations in disease biology and extent of recurrence as revealed by PSMA-PET imaging. Moreover, the marked heterogeneity of management strategies limits the interpretability of bPFS outcomes.

Importantly, the present study cannot inform on the comparative effectiveness of different PSMA-guided treatment approaches, and any such inference would be beyond the scope of the data.

Nonetheless, a discussion regarding the generalizability of our findings should also consider the different distribution and topography of potential false positive results associated with different tracers for PSMA-PET imaging. In a previous study, we observed a hierarchy between PSMA-targeted radiopharmaceuticals in differentiating the PSA nadir after therapy resulting in an impact on the oncologic outcome of patients who underwent MDT under the guidance of [^68^Ga]Ga-PSMA-11 rather than [^18^F]F-PSMA-1007 (20). In this regard, [^68^Ga]Ga-PSMA-11 has demonstrated to be characterized by a lower number of equivocal bone findings than [^18^F]F-PSMA-1007 (21). As a consequence, in the present study, patients’ management guided by [^68^Ga]Ga-PSMA-11 PET was likely associated to a lower risk of unspecific bone uptakes reducing the risk of false-positive results and subsequent suboptimal guide for SBRT.

The use of SBRT in our PSMA-positive oligometastatic population is supported by the literature evidence regarding the metachronous recurrent oligometastatic hormone-sensitive setting (20, 22–27).

In this scenario, previously published data highlighted the potential MDT benefit in terms of ADT-free survival (22) and improved outcomes, suggesting the opportunity of employing multiple rounds of MDT rather than proceeding to systemic therapy at first progression (23). However, more recent data suggest that in this setting the combination with ADT improves disease control (24, 25). In particular, the RADIOSA trial reported improved clinical PFS with the combination of SBRT and a short course of ADT (25). Nonetheless, the authors highlighted that carefully selected patients might still benefit from SBRT alone (25). Thus, in this oncological scenario, patient selection plays a pivotal role. In the quest for treatment personalization in oligometastatic hormone-sensitive disease presentations, genetic biomarkers are likely to play a critical role. Indeed, pooling STOMP (22) and ORIOLE (23), Deek et al. assessed the ability of a high-risk mutational signature to risk stratify outcomes after MDT (27). Those treated with MDT without a high-risk mutation experienced the best outcomes (median PFS 13.4 months). As opposite, a 7.5 months PFS was observed in those with a high-risk mutation treated with MDT (27). Thereafter, while individuals with oligometastatic hormone-sensitive disease without a high-risk mutation might initially be treated with MDT alone, those with a high-risk mutation need novel intensified treatment paradigms (27). In the context of treatment intensification, ARPis have emerged as a cornerstone in the management of metachronous recurrent oligometastatic hormone-sensitive PCa (28, 29). The integration of ARPis with SBRT (used as MDT) represents an area of growing clinical interest and ongoing investigation.

The phase II SATURN trial explored an intensified hormonal approach—referred to as *androgen annihilation therapy*, comprising dual ARPis—in combination with MDT among patients with oligorecurrent disease (30). Half of patients were recurrence-free 6 months after their testosterone level recovered, and less than a quarter of patients experienced a severe drug-related side effect (30).

The ongoing PERSIAN trial [NCT03449719] is currently evaluating the combination of (ADT plus) apalutamide with SBRT in the metachronous oligometastatic hormone-sensitive scenario. The early trial results, reported at ASCO GU 2025 and ESTRO 2025, suggest increased benefit for this approach in patients with <3 metastases.

On the other hand, regarding the PSMA-negative setting, these patients were mainly treated with P-Bed SRT (STD subgroup) and no concurrent HT in our study. The five-year bPFS of this subgroup was 66.8%. In the RTOG 0534 (published in 2022), the 5-year freedom from progression rates were 70.9% in group 1 (P-bed alone), 81.3% in group 2 (P-bed + short-term ADT), and 87.4% in group 3 (pelvic irradiation plus short-term ADT) (9). The RTOG 0534 subgroup analysis of baseline PSA indicated that the freedom from progression benefit observed in group 3 was greatest in patients with PSAs above the median of 0.35 ng/mL (9). Within the cluster of our PSMA-negative patients, PSA at the time of treatment was >0.35 ng/mL in 71.6% of cases (median 0.43 ng/mL). Thus, disease control in our STD patients’ subgroup would have likely been enhanced through “earlier” initiation of salvage treatment, and also a more frequent addition of ADT or the addition of pelvic irradiation (according to the RTOG 0534 data).

In terms of ADT, the use and exact duration have still to be fully defined as patient selection remains crucial (5–8). Notably, among PSMA-PET negative patients, the PSAdt assessed over the 6 months preceding imaging was ≤ 9 months in 64.6% of cases, possibly delineating a subgroup of patients with higher-risk BCR. In this setting, it has to be pointed out that intensified enzalutamide-based systemic therapy is now standard of care for high-risk BCR patients (31).

Genomic classifiers represent a contemporary tool that could help identify high-risk clusters and select patients for more individualized approaches regarding the use of systemic therapy in the postoperative salvage setting (32).

Ultimately, the key limitations of our study can be summarized as follows:

- Non-randomized design: Treatment selection was clinician-driven; outcome differences likely reflect disease biology rather than treatment effect;
- No multivariate adjustment limits the strength of conclusions;
- Prognostic—not predictive—value of PSMA-PET: it stratifies risk but does not prove benefit from PSMA-guided treatment changes;
- Heterogeneous PSMA-guided strategies: Variability in management limits bPFS interpretation and prevents comparative effectiveness conclusions;
- Small subgroup for P-bed omission: Findings are underpowered and should not guide practice outside clinical trials;
- Systemic therapy-related limitations restrict the applicability of the results to the contemporary practice: Minimal HT use with SRT in the PSMA-negative cluster; and absence of ARPIs use in PSMA-positive metastatic patients treated with SBRT-MDT (+/-HT).

Overall, the management of patients with BCR after radical prostatectomy remains challenging. Early salvage treatment is the current standard of care. Our study highlights the need for restaging with PSMA-PET to guide subsequent treatment decisions, particularly in patients with PSA levels of ≥0.5 ng/mL. At present, for PSMA-positive patients, there is a high variability of treatment options in terms of radiotherapy approaches and combinations with systemic treatments. Therefore, such options must be discussed by an experienced multidisciplinary tumor board and tailored according to the patient and disease characteristics. Finally, our study group is currently analyzing the outcomes of subsequent salvage treatments in both populations. This will be part of an upcoming secondary analysis.

## Conclusion

In nearly one-third of patients who experience prostate cancer recurrence following radical prostatectomy, PSMA-PET imaging resulted in meaningful changes in clinical management. These changes most commonly affected radiotherapy strategies — including target delineation, treatment volumes, and dose — as well as decisions regarding systemic therapy. Given the abovementioned limitations, our current study is hypothesis-generating. Emerging literature evidence suggests that treatment intensification based on PSMA-PET findings may improve patient outcomes compared to de-intensified approaches. However, the definitive impact on long-term outcomes remains to be established through ongoing large prospective randomized trials with standardized treatment algorithms and appropriate multivariate adjustment.

## Data Availability

The original contributions presented in the study are included in the article/**Supplementary Material**. Further inquiries can be directed to the corresponding author/s.

## References

[1] Freedland SJ Humphreys EB Mangold LA Eisenberger M Dorey FJ Walsh PC . Risk of prostate cancer-specific mortality following biochemical recurrence after radical prostatectomy. JAMA. (2005) 294:433–9. doi: 10.1001/jama.294.4.433, PMID: 16046649

[2] Stephenson AJ Scardino PT Eastham JA Bianco FJ Jr Dotan ZA Fearn PA . Preoperative nomogram predicting the 10-year probability of prostate cancer recurrence after radical prostatectomy. J Natl Cancer Inst. (2006) 98:715–7. doi: 10.1093/jnci/djj190, PMID: 16705126 PMC2242430

[3] Vale CL Fisher D Kneebone A Parker C Pearse M Richaud P . Adjuvant or early salvage radiotherapy for the treatment of localised and locally advanced prostate cancer: a prospectively planned systematic review and meta-analysis of aggregate data. Lancet. (2020) 396:1422–31. doi: 10.1016/S0140-6736(20)31952-8, PMID: 33002431 PMC7611137

[4] Tilki D Chen MH Wu J Huland H Graefen M D’Amico AV . Adjuvant versus early salvage radiation therapy after radical prostatectomy for pN1 prostate cancer and the risk of death. J Clin Oncol. (2022) 40:2186–92. doi: 10.1200/JCO.21.02800, PMID: 35290082 PMC9273369

[5] Parker CC Clarke NW Cook AD Kynaston H Catton CN Cross WR . Adding 6 months of androgen deprivation therapy to postoperative radiotherapy for prostate cancer: a comparison of short-course versus no androgen deprivation therapy in the RADICALS-HD randomized controlled trial. Lancet. (2024) 403:2405–15. doi: 10.1016/S0140-6736(24)00548-8, PMID: 38763154 PMC7616360

[6] Parker CC Kynaston H Cook AD Clarke NW Catton CN Cross WR . Duration of androgen deprivation therapy with postoperative radiotherapy for prostate cancer: a comparison of long-course versus short-course androgen deprivation therapy in the RADICALS-HD randomized trial. Lancet. (2024) 403:2416–25. doi: 10.1016/S0140-6736(24)00549-X, PMID: 38763153 PMC7616389

[7] Parker CC Clarke NW Cook AD Petersen PM Catton CN Cross WR . Randomised trial of no, short-term, or long-term androgen deprivation therapy with postoperative radiotherapy after radical prostatectomy: results from the three-way comparison of RADICALS-HD (NCT00541047). Eur Urol. (2024) 86:422–30. doi: 10.1016/j.eururo.2024.07.026, PMID: 39217077 PMC7617288

[8] Pollack A Pra AD . Androgen deprivation therapy combined with postoperative radiotherapy for prostate cancer management. Lancet. (2024) 403:2353–5. doi: 10.1016/S0140-6736(24)00802-X, PMID: 38763152

[9] Pollack A Karrison TG Balogh AG Gomella LG Low DA Bruner DW . The addition of androgen deprivation therapy and pelvic lymph node treatment to prostate bed salvage radiotherapy (NRG Oncology/RTOG 0534 SPPORT): an international, multicentre, randomized phase 3 trial. Lancet. (2022) 399:1886–901. doi: 10.1016/S0140-6736(21)01790-6, PMID: 35569466 PMC9819649

[10] Adebahr S Althaus A Scharl S Strouthos I Farolfi A Serani F . The prognostic significance of a negative PSMA-PET scan prior to salvage radiotherapy following radical prostatectomy. Eur J Nucl Med Mol Imaging. (2024) 51:558–67. doi: 10.1007/s00259-023-06438-3, PMID: 37736808 PMC10774185

[11] Jani AB Schreibmann E Goyal S Halkar R Hershatter B Rossi PJ . 18F-fluciclovine-PET/CT imaging versus conventional imaging alone to guide postprostatectomy salvage radiotherapy for prostate cancer (EMPIRE-1): a single centre, open-label, phase 2/3 randomized controlled trial. Lancet. (2021) 397:1895–904. doi: 10.1016/S0140-6736(21)00581-X, PMID: 33971152 PMC8279109

[12] Armstrong WR Kishan AU Booker KM Grogan TR Elashoff D Lam EC . Impact of prostate-specific membrane antigen positron emission tomography/computed tomography on prostate cancer salvage radiotherapy management: results from a prospective multicenter randomized phase 3 trial (PSMA-SRT NCT03582774). Eur Urol. (2024) 86:52–60. doi: 10.1016/j.eururo.2024.10.022, PMID: 38290964 PMC12882065

[13] Calais J Ceci F Eiber M Hope TA Hofman MS Rischpler C . 18F-fluciclovine PET-CT and 68Ga-PSMA-11 PET-CT in patients with early biochemical recurrence after prostatectomy: a prospective, single-centre, single-arm, comparative imaging trial. Lancet Oncol. (2019) 20:1286–94. doi: 10.1016/S1470-2045(19)30593-5, PMID: 31375469 PMC7469487

[14] Deandreis D Guarneri A Ceci F Lillaz B Bartoncini S Oderda M . 68Ga-PSMA-11 PET/CT in recurrent hormone-sensitive prostate cancer (HSPC): a prospective single-centre study in patients eligible for salvage therapy. Eur J Nucl Med Mol Imaging. (2020) 47:2804–15. doi: 10.1007/s00259-020-04809-8, PMID: 32314028

[15] Ceci F Rovera G Iorio GC Guarneri A Chiofalo V Passera R . Event-free survival after 68 Ga-PSMA-11 PET/CT in recurrent hormone-sensitive prostate cancer (HSPC) patients eligible for salvage therapy. Eur J Nucl Med Mol Imaging. (2022) 49:3257–68. doi: 10.1007/s00259-022-05741-9, PMID: 35217883 PMC9250462

[16] Cornford P Bellmunt J Bolla M Briers E De Santis M Gross T . Part II: treatment of relapsing, metastatic, and castration-resistant prostate cancer. Eur Urol. (2017) 71:630–42. doi: 10.1016/j.eururo.2016.08.002, PMID: 27591931

[17] Ost P Siva S Brabrand S Dirix P Liefhooghe N Otte FX . Salvage metastasis-directed therapy versus elective nodal radiotherapy for oligorecurrent nodal prostate cancer metastases (PEACE V-STORM): a phase 2, open-label, randomized controlled trial. Lancet Oncol. (2025) 26:695–706. doi: 10.1016/S1470-2045(25)00197-4, PMID: 40339593

[18] Slevin F Alexander S Brown SR Carter M Choudhury A Clipson A . Pelvis Or Involved Node Treatment: Eradicating Recurrence in Prostate Cancer (POINTER-PC) - study protocol paper for a phase III multicentre, open-label randomized controlled trial. BMJ Open. (2024) 14:e095560. doi: 10.1136/bmjopen-2024-095560, PMID: 39725427 PMC11683931

[19] Belliveau C Saad F Duplan D Petit C Delouya G Taussky D . Prostate-specific membrane antigen PET-guided intensification of salvage radiotherapy after radical prostatectomy: A phase 2 randomized clinical trial. JAMA Oncol. (2025) 11:1431–8. doi: 10.1001/jamaoncol.2025.3746, PMID: 41037308 PMC12581501

[20] Bauckneht M Lanfranchi F Albano D Triggiani L Linguanti F Urso L . Diverse imaging methods may influence long-term oncologic outcomes in oligorecurrent prostate cancer patients treated with metastasis-directed therapy (the PRECISE-MDT study). J Nucl Med. (2024) 65:1202–9. doi: 10.2967/jnumed.124.267586, PMID: 38906557 PMC11294064

[21] Rizzo A Morbelli S Albano D Fornarini G Cioffi M Laudicella R . The Homunculus of unspecific bone uptakes associated with PSMA-targeted tracers: a systematic review-based definition. Eur J Nucl Med Mol Imaging. (2024) 51:3753–64. doi: 10.1007/s00259-024-06797-5, PMID: 38884773 PMC11445318

[22] Ost P Reynders D Decaestecker K Fonteyne V Lumen N De Bruycker A . Surveillance or metastasis-directed therapy for oligometastatic prostate cancer recurrence: A prospective, randomized, multicenter phase II trial. J Clin Oncol. (2018) 36:446–53. doi: 10.1200/JCO.2017.75.4853, PMID: 29240541

[23] Phillips R Shi WY Deek M Radwan N Lim SJ Antonarakis ES . Outcomes of observation vs stereotactic ablative radiation for oligometastatic prostate cancer: the ORIOLE phase 2 randomized clinical trial. JAMA Oncol. (2020) 6:650–9. doi: 10.1001/jamaoncol.2020.0147, PMID: 32215577 PMC7225913

[24] Tang C Sherry AD Haymaker C Bathala T Liu S Fellman B . Addition of metastasis-directed therapy to intermittent hormone therapy for oligometastatic prostate cancer: the EXTEND phase 2 randomized clinical trial. JAMA Oncol. (2023) 9:825–34. doi: 10.1001/jamaoncol.2023.0161, PMID: 37022702 PMC10080407

[25] Marvaso G Corrao G Zaffaroni M Vincini MG Lorubbio C Gandini S . ADT with SBRT versus SBRT alone for hormone-sensitive oligorecurrent prostate cancer (RADIOSA): a randomized, open-label, phase 2 clinical trial. Lancet Oncol. (2025) 26:300–11. doi: 10.1016/S1470-2045(24)00730-7, PMID: 40049196

[26] Lanfranchi F Belgioia L Albano D Triggiani L Linguanti F Urso L . Impact of metastasis-directed therapy guided by different PET/CT radiotracers on distant and local disease control in oligorecurrent hormone-sensitive prostate cancer: A secondary analysis of the PRECISE-MDT study. Radiol Imaging Cancer. (2025) 7:e240150. doi: 10.1148/rycan.240150, PMID: 40377422 PMC12130722

[27] Deek MP van der Eecken K Sutera P Deek RA Fonteyne V Mendes AA . Long-term outcomes and genetic predictors of response to metastasis-directed therapy versus observation in oligometastatic prostate cancer: analysis of STOMP and ORIOLE trials. J Clin Oncol. (2022) 40:3377–82. doi: 10.1200/JCO.22.00644, PMID: 36001857 PMC10166371

[28] Gillessen S Turco F Davis ID Efstathiou JA Fizazi K James ND . Management of patients with advanced prostate cancer. Report from the 2024 advanced prostate cancer consensus conference (APCCC). Eur Urol. (2025) 87:157–216. doi: 10.1016/j.eururo.2024.09.017, PMID: 39394013

[29] Chi KN Agarwal N Bjartell A Chung BH Pereira de Santana Gomes AJ Given R . Apalutamide for metastatic, castration-sensitive prostate cancer. N Engl J Med. (2019) 381:13–24. doi: 10.1056/NEJMoa1903307, PMID: 31150574

[30] Nikitas J Rettig M Shen J Reiter R Lee A Steinberg ML . Systemic and tumor-directed therapy for oligorecurrent metastatic prostate cancer (SATURN): primary endpoint results from a phase 2 clinical trial. Eur Urol. (2024) 85:517–20. doi: 10.1016/j.eururo.2024.01.021, PMID: 38494380 PMC11386258

[31] Freedland SJ de Almeida Luz M De Giorgi U Gleave M Gotto GT Pieczonka CM . Improved outcomes with enzalutamide in biochemically recurrent prostate cancer. N Engl J Med. (2023) 389:1453–65. doi: 10.1056/NEJMx250003, PMID: 37851874

[32] Dal Pra A Ghadjar P Hayoz S Liu VYT Spratt DE Thompson DJS . Validation of the Decipher genomic classifier in patients receiving salvage radiotherapy without hormone therapy after radical prostatectomy - an ancillary study of the SAKK 09/10 randomized clinical trial. Ann Oncol. (2022) 33:950–8. doi: 10.1016/j.annonc.2022.05.007, PMID: 35636621

