# Supplementary material for: PSMA-guided management of recurrent post-prostatectomy patients: a sub-analysis of a prospective single-center study
Source: Front Oncol. 2026 Jan 29;16:1764588. doi: 10.3389/fonc.2026.1764588 (PMC12893980; doi:10.3389/fonc.2026.1764588)
Supplement: Supplementary file 1 [file Table1.docx]

| Radiotherapy Details |  |
| --- | --- |
| SRT Dose (n = 75)  70 Gy / 35 fx  52.5 Gy / 20 fx  74 Gy / 37 fx* | n = 62 (82.6%) n = 11 (14.7%) n = 2 (2.7%) |
| SRT Technique (n = 75)  3D-CRT   VMAT | n = 64 (85.3%) n = 11 (14.7%) |
| SBRT Dose (n = 14)  35 Gy / 5 fx  16 Gy / 1 fx | n = 12 (85.7%)  n = 2 (14.3%) |

Supplementary Table 1. Radiotherapy Details (overall population).

*Notes: SRT, salvage radiotherapy; SBRT, stereotactic body radiotherapy.*

**Dose-escalation approach for PSMA-positive patients with a prostate bed uptake.*

| SBRT toxicity | G1 | G2 | G3 | G4 | G5 |
| --- | --- | --- | --- | --- | --- |
| Acute GU toxicity | 1 | 0 | 0 | 0 | 0 |
| Acute GI toxicity | 0 | 0 | 0 | 0 | 0 |
| Acute pain toxicity | 0 | 0 | 0 | 0 | 0 |
| Late GU toxicity | 1 | 0 | 0 | 0 | 0 |
| Late GI toxicity | 0 | 0 | 0 | 0 | 0 |
| Late pain toxicity | 0 | 0 | 1 | 0 | 0 |

Supplementary Table 2. SBRT-MDT related toxicity according to CTCAE v4.03.

*Notes: GU, genitourinary; GI, gastrointestinal; SBRT, stereotactic body radiotherapy; MDT, metastases-directed therapy.*
